# Reduction of Mitophagy-Related Oxidative Stress and Preservation of Mitochondria Function Using Melatonin Therapy in an HT22 Hippocampal Neuronal Cell Model of Glutamate-Induced Excitotoxicity

**DOI:** 10.3389/fendo.2019.00550

**Published:** 2019-08-08

**Authors:** Dan-dan Wang, Mei-fang Jin, Dong-jing Zhao, Hong Ni

**Affiliations:** Division of Brain Science, Institute of Pediatric Research, Children's Hospital of Soochow University, Suzhou, China

**Keywords:** melatonin, mitochondria, mitophagy, glutamate, HT22

## Abstract

Recent evidence indicates that autophagy-mediated mitochondrial homeostasis is crucial for oxidative stress-related brain damage and repair. The highest concentration of melatonin is in the mitochondria of cells, and melatonin exhibits well-known antioxidant properties. We investigated the impact and mechanism involved in mitochondrial function and the mitochondrial oxidative stress/autophagy regulator parameters of glutamate cytotoxicity in mouse HT22 hippocampal neurons. We tested the hypothesis that melatonin confers neuroprotective effects via protecting against mitochondrial impairment and mitophagy. Cells were divided into four groups: the control group, melatonin alone group, glutamate injury group, and melatonin pretreatment group. We found that glutamate induced significant changes in mitochondrial function/oxidative stress-related parameters. Leptin administration preserved mitochondrial function, and this effect was associated with increased superoxide dismutase, glutathione (GSH), and mitochondrial membrane potential and decreased GSSG (oxidized glutathione) and mitochondrial reactive oxygen species. Melatonin significantly reduced the fluorescence intensity of mitophagy via the Beclin-1/Bcl-2 pathway, which involves Beclin-1 and Bcl-2 proteins. The mitophagy inhibitor CsA corrected these glutamate-induce changes, as measured by the fluorescence intensity of Mitophagy-Tracker Red CMXROS, mitochondrial ROS, and mitochondrial membrane potential changes. These findings indicate that melatonin exerts neuroprotective effects against glutamate-induced excitotoxicity by reducing mitophagy-related oxidative stress and maintaining mitochondrial function.

## Introduction

The developing brain undergoes constant maturation. The developing brain exhibits higher brain excitability, and it is more prone to convulsions than the adult brain. The incidence of neonatal seizures is 1.1–8.6/1,000 live births (1). Status epilepticus (SE) occurs more frequently in children than adults, and 40–50% occur in children under 2 years of age (2). Gluckman et al. (3) reported that ~75% of neonates with hypoxic ischemic encephalopathy (HIE) developed seizures, and some of these children developed epilepsy in adulthood. Conventional antiepileptic drugs that are currently used in clinical practice have adverse side effects, such as white matter damage, in convulsive children caused by HIE (4). To make things worse, ~20% of children with epilepsy develop drug-resistant epilepsy (DRE), in which seizures cannot be controlled with further manipulation of antiepileptic drugs (5). Phenobarbital (PB) is a first-line antiepileptic drug (AED) for the treatment of neonatal seizures, but it produces long-term cognitive and intellectual impairments in children with febrile seizures (6). Therefore, there is still a need to better understand the molecular mechanisms underlying the effects of developmental seizures on brain maturation and dysfunction before optimizing age-appropriate treatments.

Unlike traditional antiepileptic drugs, significant progress was made recently in understanding the effects and mechanisms of endocrine modulators, such as melatonin, leptin and ghrelin, on pediatric neurological diseases, particularly epilepsy (7–10). The ketogenic diet (KD) has a history of nearly 100 years of success in the treatment of epilepsy that is refractory to antiepileptic drugs. However, a KD also affects children's normal growth and development, especially brain development (11). The mechanisms of action of KD are poorly defined but certainly involve a change in metabolism. A KD modulates leptin, ghrelin, and melatonin plasma levels and gene expression in the brain, which suggests that these endocrine regulatory molecules have neuroprotective or antiepileptic effects in epilepsy and serve as potential markers of antiepileptic drug responses (12, 13). Clinical and experimental results show that melatonin reduces sleep disorders and circadian rhythm changes alone, and it reduces seizures in combination with AEDs (14). Therefore, it may be used as an adjuvant treatment for epilepsy to lower cost and reduce toxicity. Intensive research by our group and others demonstrated that melatonin was beneficial in experimental models of brain damage caused by developmental seizures (15–17). However, previous research, including our own work, was primarily performed using an *in vivo* animal model, but there are very few studies using cell models. Only three studies on Pubmed used a cultured hippocampal neuron excitotoxic cell model to examine the neuroprotective effects and mechanisms of melatonin. Quiros et al. (18) showed that melatonin reduced glucocorticoid-induced toxicity in hippocampal HT22 cells in the presence of neurotoxins. Herrera et al. (19) demonstrated that melatonin prevented mitochondrial ROS production *in vitro* against glutamate-induced oxytosis in the HT22 mouse hippocampal cell line. Lezoualc'h et al. (20) used cells of the clonal hippocampal cell line HT22 and organotypic hippocampal rat brain slice cultures and found that melatonin protected HT22 cells and organotypic hippocampal slices from glutamate- and H_2_O_2_-induced cell death. We recently demonstrated that mitophagy-mediated mitochondrial activation contributed to glutamate-induced HT22 neuronal cell damage, and leptin treatment counteracted these adverse effects. However, whether the antioxidant and neuroprotective abilities of melatonin in glutamate-induced *in vitro* neuronal injury is dependent on the same signaling pathway is not known.

The highest concentration of melatonin is in the mitochondria of cells, and it has well-known antioxidant properties (21). Nopparat et al. (22) demonstrated that melatonin protected methamphetamine-induced cell death via by inhibition of Bcl-2/Beclin-1 mediated autophagy. Melatonin inhibited hypoxia-mediated mitophagy in human hepatocarcinoma cells (23). We recently demonstrated that melatonin induced long-term expression changes of energy metabolism-related genes in the hippocampus, including Kcnj11, leptin receptor, dopamine receptor D2, melanocortin 4 receptor, ACAT1, and Cathepsin-E, in a rat model of brain injury induced by neonatal seizures (24, 25). These genes are highly associated with mitochondrial function and autophagy. Therefore, we hypothesized that melatonin provides beneficial effects on mitochondrial function via reducing autophagy and, more specifically, mitophagy.

The present study investigated mitochondrial function, mitochondrial oxidative stress, and mitophagy parameters using an *in vitro* model of glutamate-induced cytotoxicity in mouse HT22 hippocampal neurons. Cell viability, parameters of mitochondrial function and oxidative stress, and biomarkers for mitophagy, including Bcl-2/Beclin-1 protein levels, were measured.

## Materials and Methods

### Cell Lines

The HT22 mouse hippocampal neuronal cell line was purchased from the cell bank of Institute of Cell Biology, Chinese Academy of Sciences.

### Reagents and Antibodies

DMEM/high glucose medium and the protein molecular weight marker were purchased from Thermo Fisher Scientific Company (USA). Fetal bovine serum (FBS) was purchased from Serana Company (Germany). L-glutamate, cyclosporine A (CsA), and melatonin were purchased from Sigma-Aldrich Company (USA). The BCA Protein Assay Kit and RIPA lysis buffer were purchased from Beyotime Institute of Biotechnology. An antibody against β-actin was purchased from Sigma-Aldrich. Primary antibodies against Beclin-1 were purchased from Cell Signaling Technology (USA). Antibodies against Bcl-2 and secondary antibodies for immunoblots were HRP-conjugated anti-rabbit and anti-mouse IgGs from Santa Cruz Biotechnology (USA).

### Cell Culture Conditions

HT22 cells were cultured in DMEM supplemented with 10% fetal bovine serum, 100 U/ml of penicillin, and 100 mg/ml of streptomycin in humidified air at 37°C with 5% CO_2_.

### Drug Treatment and Grouping

Cells were seeded into each well of a 6-well or 96-well plate the day before the experiment. The control group (Control), melatonin alone group (Melatonin), glutamate injury group (Glutamate) and melatonin pretreatment group (Glutamate + Melatonin) were established. Melatonin in the melatonin alone group was added to the culture medium to obtain a final concentration at 10^−7^ mol/l. Glutamate in the glutamate injury group was added to the culture medium to obtain a final concentration at 5 mM (26). Cells in the melatonin pretreatment group were pretreated with 10^−7^ mol/l melatonin in culture for 2 h before glutamate was added to the medium to obtain a final concentration at 5 mM. Cells were subjected to various measurements as described below after further incubation for 24 h.

### Cell Viability Assay

Cells were seeded at a cell density of 5 × 10^3^ cells/well in 96-well tissue culture plates. After various treatments, we switched the medium in each well with 100 μl DMEM medium containing 10% CCK-8 Cell Proliferation Reagent Cell Counting Kit-8 (CCK8) (5 mg/ml) (Dojindo Molecular Technologies, Kumamoto, Japan) and incubated for an additional 2 h. The absorbance (OD) of the samples was measured at 450 nm using a 96-well plate reader (27).

### Lactate Dehydrogenase (LDH) Assay

The LDH release assay was used to determine membrane integrity. The cell density was adjusted to a concentration of 5 × 10^4^, and the cells were cultured in 96-well plates with 100 μl per well. After treatment, 50 μl of supernatant from each well was collected and incubated with the same volume of reaction mixture from the LDH cytotoxicity detection kit (Nanjing Jiancheng Bioengineering Institute, Nanjing, China). The activity of LDH was calculated from the absorbance at 440 nm in a multi-functional microporous plate reader (28, 29).

### Biochemical Analysis of Oxidative Stress Markers

Cells in the logarithmic phase were collected and cultured in 6-well plates at 2 × 10^5^ cells/well. After treatment, the cells were lysed in 200 μL RIPA buffer. Cells were lysed on ice for 30 min, and proteins were crushed using an ultrasonic cell crusher. After centrifugation for 10 min at 12,000 rpm, the supernatant was deproteinated using a buffer solution containing metaphosphoric acid (10% w/v) and triethanolamine (53.1% v/v) to remove proteins and avoid interference from protein-sulfhydryl groups. SOD, GSH, and GSSG levels were measured using SOD, GSH, and GSSG assay kits (Nanjing Jiancheng Bioengineering Institute, Nanjing, China) in accordance with the manufacturer's instructions, and the absorbance of each group was measured using a microplate reader (30, 31).

### Analysis of Mitochondrial Membrane Potentials Using Mito-Tracker

Mito-Tracker Green FM and Mito-Tracker Red CMXROS were included in the mitochondrial fluorescence probe Mito-Tracker kit (Invitrogen, USA). Mito-Tracker Green FM is a mitochondrial green fluorescent dye that locates stable mitochondria, and it is not affected by mitochondrial membrane potential. The Mito-Tracker Red CMXROS probe is a red fluorescent dye that is passively transported through the cell membrane and directly assembled in active mitochondria. The accumulation of this dye depends on the mitochondrial membrane potential. Cells in logarithmic growth phase were collected and cultured in 6-well plates. Cells were collected after treatment, digested with trypsinase, washed three times with serum-free medium, and re-suspended in a mixed solution of 0.5 ml 200 nM Mito-Tracker Green FM and 25 nM Mito-Tracker Red CMXROS for a 30-min incubation at 37°C in darkness. Cells were washed three times with PBS containing 1% serum, and re-suspended in an appropriate amount of PBS containing 1% serum. A Gallios flow cytometer (Beckman Coulter, Brea, CA, USA) was used to detect the fluorescence ratio of the FL-1 channel (green fluorescence) and FL-2 channel (red fluorescence) and determine the changes of mitochondrial membrane potential. The data were analyzed using FlowJo Analysis Software.

Another group of HT22 cells were seeded in confocal dishes and cultured overnight. Cells were washed three times with serum-free medium after treatment, and a mixed solution of 500 μl prewarmed 200 nM Mito-Tracker Green FM and 25 nM Mito-Tracker Red CMXROS was added. Cells were incubated in an incubator for 30 min, washed three times with serum-free medium and provided with fresh medium. Confocal fluorescence microscopy was used to observe the results of mitochondrial staining, and photographs were taken (32, 33).

### Analysis of Mitochondrial Membrane Potentials Using JC-1

Cells were stained after treatment with the mitochondrial membrane potential sensitive probe JC-1 (Beyotime, Shanghai, China). Cells were incubated in an incubator for 30 min at a final concentration of 2 μM in PBS and washed twice with PBS. JC-1 aggregate was measured at the FL-2 channel, and green fluorescence (JC-1 monomer) was measured at the FL-1 channel. The data were analyzed using the FlowJo analysis software, and the results are displayed in a dot plot of J-aggregate red fluorescence (y-axis) against JC-1 green fluorescence (x-axis) (34).

### Determination of Mitochondria Reactive Oxygen Species (ROS) (MitoSOX)

MitoSOX^TM^ (Thermo Fisher Scientific, Waltham, MA, USA) Red Reagent penetrates into living cells and produces red fluorescence under oxidative damage of mitochondria. Cells were collected after treatment, washed with HBSS three times and suspended in a mixed solution of 0.5 ml 5 μM MitoSOX and 200 nM Mito-Tracker Green FM. Cells were incubated for 30-min in an incubator, washed with HBSS three times, and suspended in an appropriate amount of HBSS. The red-green fluorescence ratio was measured using a Gallios flow cytometer.

Another group of cells in logarithmic phase were cultured in 6-well plates at 2 × 10^5^ cells per well. The medium was discarded after treatment, and cells were washed with HBSS three times. One milliliter of a 5-μM MitoSOX solution was added, and cells were incubated at 37°C for 10 min. Cells were washed with HBSS three times, stained with 1 μg/ml Hoechst 33342 for 10 min, washed with HBSS three times and provided with fresh medium. Confocal fluorescence microscopy was used to observe and take photographs for analysis (35–37).

### Mitophagy Assay

The Mitophagy Detection Kit (Dojindo Molecular Technologies, Kumamoto, Japan) contained Mitophagy Dye and Lyso Dye, which were used to monitor mitophagy. Mitochondrial autophagosome fuse with lysosomes during mitophagy, and the fluorescence intensity of Mtphagy Dye increases. Cells in logarithmic phase were seeded in confocal dishes and cultured overnight. The cells were washed with Hanks-HEPES buffer 3 times and incubated with 500 μl of 100 nmol/l Mtphagy Dye solution for 30 min. Cells were washed and treated with glutamate and/or melatonin. The medium was removed after a 24-h culture, and cells were incubated with 500 μl of a 1-μmol/l Lyso Dye solution at 37°C in the absence of light. Cells were washed three times, and the co-localization of Mtphagy and Lyso Dyes was observed using confocal fluorescence microscopy (38).

### Western Blot Assay

Treated cells were retrieved, and an appropriate amount of lysis buffer was added for lysing on ice for 30 min. An ultrasonic crusher was used to ensure complete crushing of proteins, and the supernatant was recovered after 12,000 r/min centrifugation for 30 min. A BCA protein quantitative kit was used to determine the amount of protein in each sample group. Proteins were boiled for 10 min in the sample buffer, and samples were loaded for electrophoresis. Proteins were transferred to a PVDF membrane, which was incubated in a blocking solution at room temperature for 2 h. Beclin-1 and Bcl-2 antibodies were incubated overnight at 4°C, and the secondary antibody was incubated at room temperature for 1 h. Proteins were imaged using chemiluminescent autography. The strip optical density of the image was formed and analyzed using image analysis software.

### Cell Culture With Cyclosporine A and Mitochondrial Function Detection

Cyclosporine A (CsA) (a mitophagy inhibitor) was used to observe the effect of glutamate on mitophagy. Cells were divided into a glutamate group and cyclosporine A intervention group. The cyclosporine A intervention group received 0.5 μM CsA 2 h before glutamate followed by 24 h in culture. The function of mitochondria was observed using JC-1, Mito-Tracker and MitoSOX assay kits, and the level of mitophagy was determined using a mitophagy detection kit and Western blotting (39).

### Statistical Analysis

GraphPad Prism version 5.0 was used for data processing. The experimental results are expressed in means ± SEM. One-way ANOVA followed by bonferoni *post-hoc* analysis was used to compare the means of more than two groups. A *P*-value < 0.05 indicated that the difference was statistically significant.

## Results

### Effects of Melatonin on Glutamate-Induced Morphological Changes

According to the relevant literature (40, 41) and our preliminary experiments, we used different concentrations of melatonin (10^−4^, 10^−5^, 10^−6^, 10^−7^, 10^−8^, and 10^−9^ M) for efficacy tests. We found that 10^−7^ M melatonin significantly increased cell viability (at 5 mmol/ml glutamate). We further examined the effect of melatonin alone at a concentration of 10^−7^ M on cell survival and found that this concentration produced no cytotoxic effects on the cells. Therefore, 10^−7^ M melatonin was used for the experimental studies.

**Figure 1:**
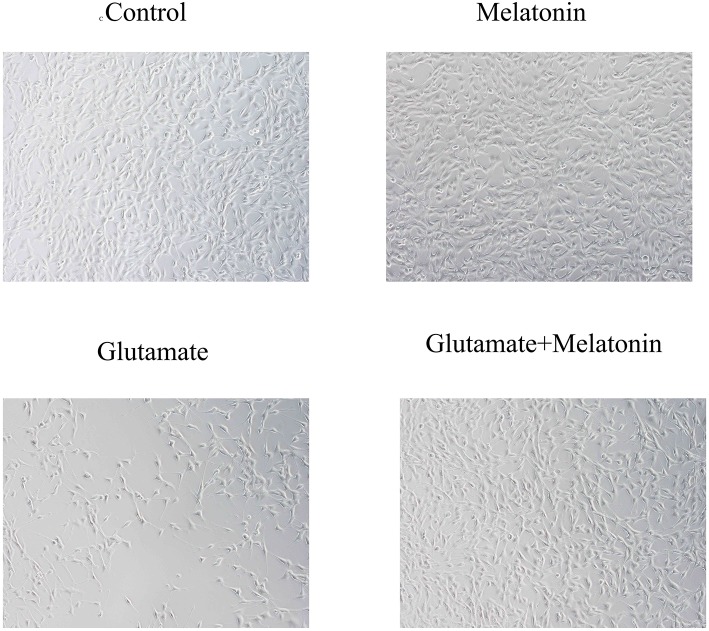
Observation of cell morphology in each group under light microscopy (×10).

Light microscopy revealed that cells in the control group adhered to the wall, exhibited a round nucleus, a smooth and intact cell membrane, obvious protrusions and synapses that were interwoven into a network. There was no significant difference between the melatonin alone group and the control group. Cells in the glutamate injury group exhibited weak adherence, significantly inhibited cell proliferation, smaller cell volume, concentrated nuclei, an interrupted synaptic network between cells, and increasingly shorter protrusions. Cells in the melatonin pretreatment group exhibited restored normal morphology and characteristics of obvious proliferation and established synaptic connections between cells. These results demonstrated that melatonin inhibited cell necrosis and apoptosis induced by glutamate neurotoxicity (Figure 1).

### Evaluation of the Protective Effect of Melatonin on HT22 Cells Using the CCK8 Assay and LDH Release Assay

**Figure 2:**
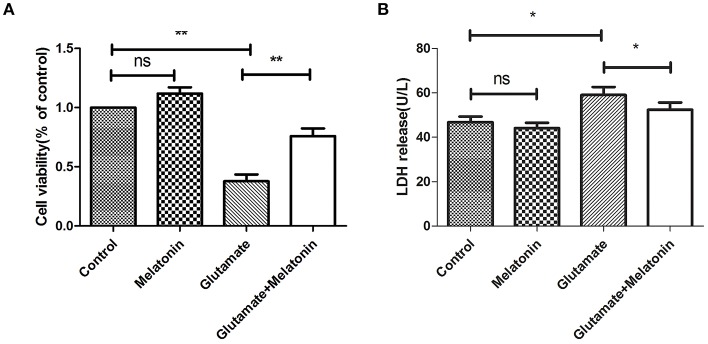
Protective effect of melatonin on glutamate-induced injury in HT22 cells. **(A)** Effect of melatonin on cell survival rate: *F* = 27.242, *P* < 0.001; control group vs. glutamate injury group: *P* < 0.001, glutamate injury group vs. melatonin pretreatment group: *P* = 0.005; **(B)** Effect of melatonin on the release of LDH: *F* = 11.553, *P* < 0.001; control group vs. glutamate injury group: *P* = 0.011, glutamate injury group vs. melatonin pretreatment group: *P* = 0.039; *P < 0.05, **P < 0.01. ns, not significant (*n* = 6/group).

The well-validated survival assay (CCK-8) demonstrated that exposure of HT22 cells to 5 mM L-glutamine for 24 h elicited maximum cell death. The results show that glutamate induced an obvious decrease in cell viability, and cell viability in the melatonin pretreatment group rebounded significantly (Figure 2A).

LDH release was also measured. An increase in LDH release into the culture medium suggests cell injury. The LDH release in melatonin alone group was unchanged in the present study, but it increased in the glutamate injury group. LDH release in the melatonin pretreatment group decreased compared to the glutamate group, which is consistent with the CCK8 assay results (Figure 2B). These results suggest that melatonin protects HT22 cells from glutamate-induced damage.

### Effect of Melatonin on Glutamate-Induced Oxidative Stress in HT22 Cells

**Figure 3:**
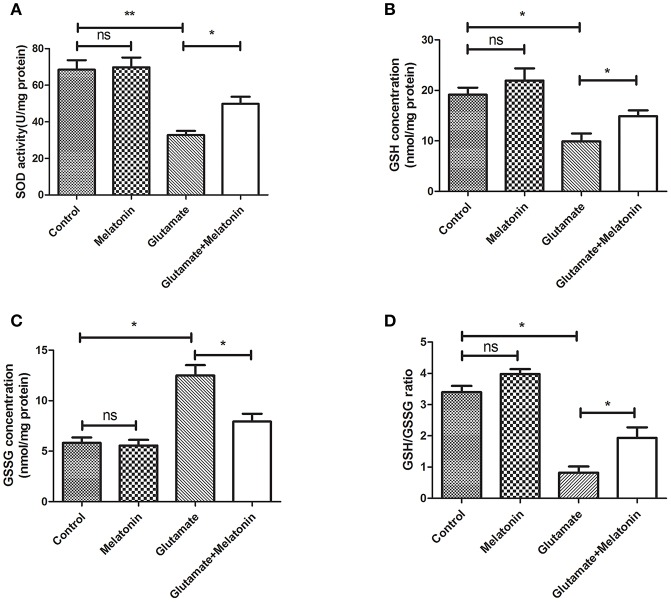
Effect of melatonin on glutamate-induced oxidation of HT22 cells. **(A)** Effect of melatonin on the activity of SOD in cells: *F* = 19.937, *P* < 0.001; control group vs. glutamate injury group: *P* < 0.001, glutamate injury group vs. melatonin pretreatment group: *P* = 0.034; **(B)** Effect of melatonin on the intracellular GSH concentration: *F* = 9.315, *P* < 0.001; control group vs. glutamate injury group: *P* = 0.011, glutamate injury group vs. melatonin pretreatment group: *P* = 0.044; **(C)** Effect of melatonin on the intracellular GSSG concentration: *F* = 5.921, *P* = 0.005; control group vs. glutamate injury group: *P* = 0.014, glutamate injury group vs. melatonin pretreatment group: *P* = 0.045; **(D)** Effects of melatonin on GSH/GSSG ratio in cells: *F* = 8.363, *P* = 0.001; control group vs. glutamate injury group: *P* = 0.011, glutamate injury group vs. melatonin pretreatment group: *P* = 0.039; *P < 0.05, **P < 0.01. ns, not significant (*n* = 6/group).

The levels of SOD, GSH, GSSG, and GSH/GSSG in each group were measured. SOD activity, GSH concentration and the GSH/GSSG ratio in the glutamate injury group decreased sharply, and GSH concentration increased significantly (Figures 3A,B,D). The differences between the glutamate injury group, the control group and the melatonin pretreatment group were highly statistically significant (Figure 3C). These results suggest that melatonin reversed the changes in these oxidative indicators induced by glutamate and maintained normal cellular oxidative levels in cells.

### Effect of Melatonin on Glutamate-Induced Mitochondrial Membrane Potential Changes Using Mito-Tracker

**Figure 4:**
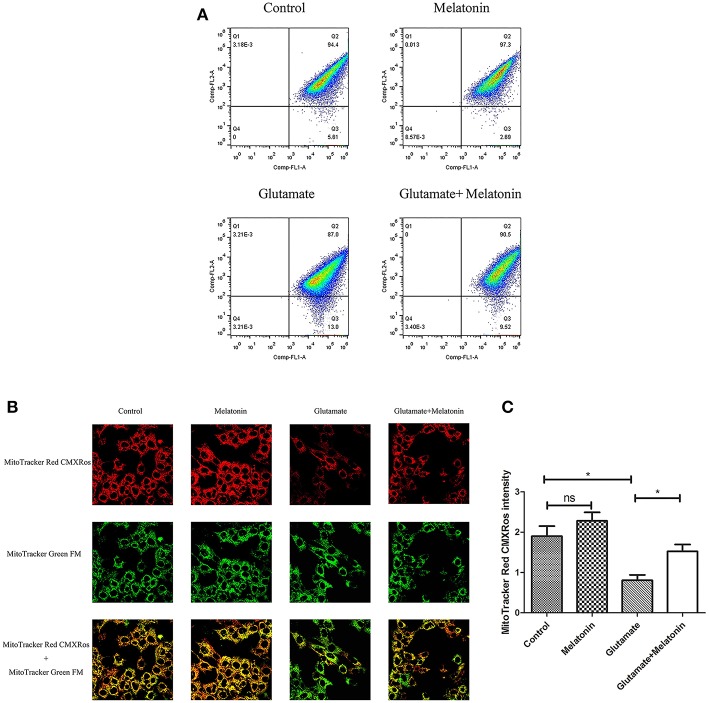
Effects of melatonin on glutamate-induced mitochondrial membrane potential changes by Mito-Tracker. **(A)** Detection of cell mitochondrial membrane potential changes by flow cytometry; **(B)** Fluorescence intensity of Mito-Tracker Red CMXROS in cells; **(C)** Quantitative analysis of mean fluorescence intensity of Mito-Tracker Red CMXROS in cells: *F* = 10.946, *P* < 0.001; control group vs. glutamate injury group: *P* = 0.011, glutamate injury group vs. melatonin pretreatment group: *P* = 0.035; *P < 0.05. ns, not significant (*n* = 6/group).

The fluorescence ratios of the FL-1 channel (green fluorescence) and FL-2 channel (red fluorescence) were measured using flow cytometry. The results showed that the number of red monosomic cells decreased in the glutamate injury group, and increased after melatonin intervention, which indicates that melatonin improved the glutamate-induced migration of the cell population (Figure 4A).

Changes of mitochondrial membrane potential in HT22 cells were observed under confocal microscopy. The red fluorescence intensity of Mito-Tracker Red CMXROS in the glutamate injury group declined greatly. Melatonin restored the red fluorescence intensity in the melatonin pretreatment group (Figures 4B,C). These results indicate that glutamate decreased mitochondrial membrane potential, and melatonin significantly maintained the normal level of mitochondrial membrane potential.

### Effect of Melatonin on Glutamate-Induced Mitochondrial Membrane Potential Changes Using JC-1

**Figure 5:**
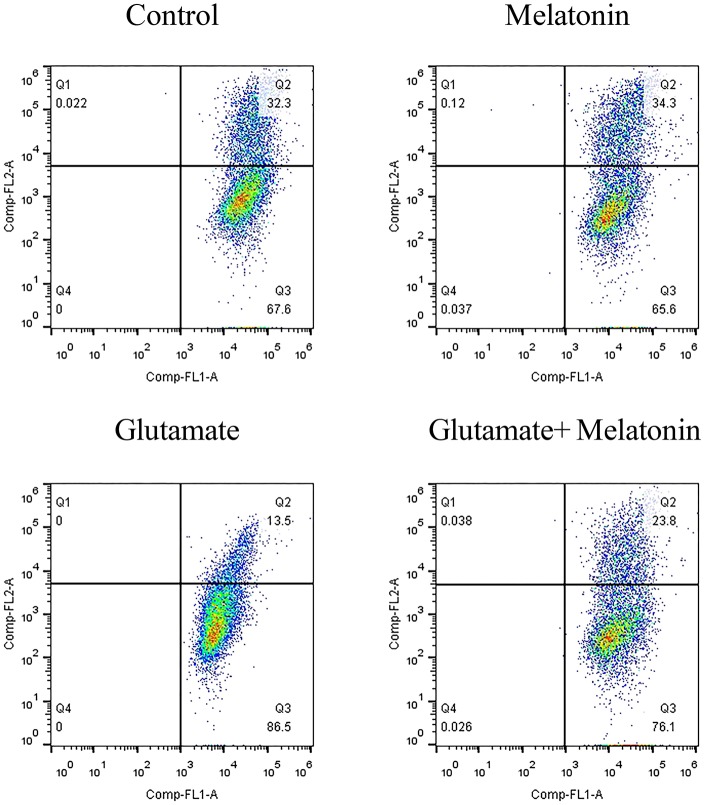
Effect of melatonin on glutamate-induced mitochondrial membrane potential changes using JC-1.

When the mitochondrial membrane potential is relatively high, JC-1 aggregates in the matrix of mitochondria to form a polymer that produces red fluorescence. When the mitochondrial membrane potential is low, JC-1 cannot aggregate in the matrix of mitochondria, which produces green fluorescence. The decrease in mitochondrial membrane potential is a marker event in the early stage of apoptosis. Therefore, the change of JC-1 from red to green fluorescence is an indicator of early stage apoptosis. The results of flow cytometry showed that the number of green monosomic cells increased in the glutamate injury group, i.e., the mitochondrial membrane potential decreased, and the mitochondrial membrane potential increased in the melatonin pretreatment group (Figure 5). These results are consistent with the results of the mitochondrial membrane potential results using the mitochondrial fluorescence probe Mito-Tracker. These results indicate that melatonin reversed the glutamate-induced decrease in mitochondrial membrane potential and inhibited apoptosis.

### Effect of Melatonin on Glutamate-Induced Mitochondrial ROS Accumulation in HT22 Cells

**Figure 6:**
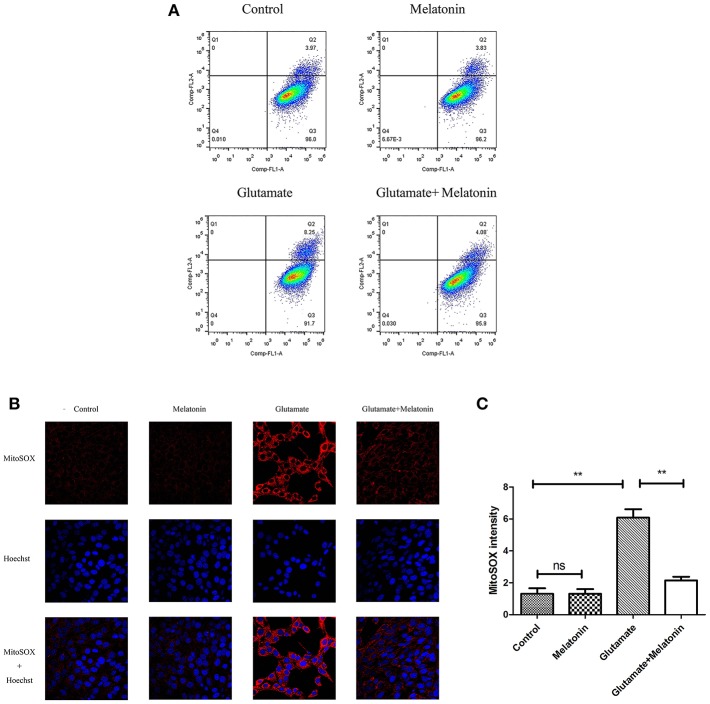
Effect of melatonin on mitochondrial reactive oxygen species (ROS) accumulation in cells. **(A)** Detection of ROS content in mitochondria using flow cytometry; **(B)** Fluorescence intensity of ROS in mitochondria; **(C)** Quantitative analysis of average fluorescence intensity of ROS in mitochondria: *F* = 24.903, *P* < 0.001; control group vs. glutamate injury group: *P* < 0.001, glutamate injury group vs. melatonin pretreatment group: *P* < 0.001; **P < 0.01. ns, not significant (*n* = 6/group).

Flow cytometry revealed an increase in red monosomic cells in the glutamate injury group. Red monosomic cells decreased in the melatonin pretreatment group compared to the glutamate group (Figure 6A).

Confocal microscopy indicated significantly higher levels of mitochondrial ROS content in the glutamate injury group than the control group, which indicates that glutamate enhanced the production of ROS in mitochondria. Melatonin pretreatment reduced the accumulation of mitochondrial ROS content (Figures 6B,C). These results demonstrate that melatonin inhibited the glutamate-induced increase in ROS content in cells and protected the cells.

### Effect of Melatonin on Glutamate-Induced Mitochondrial Autophagy in HT22 Cells

**Figure 7:**
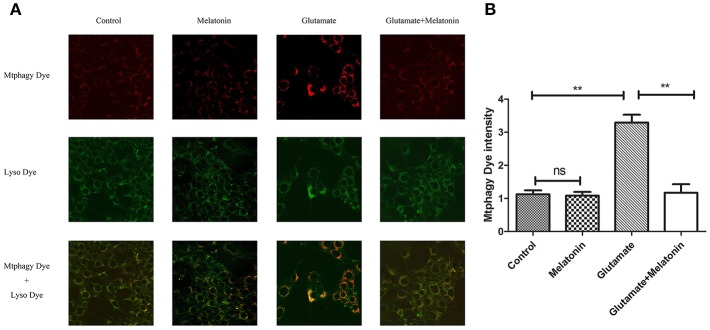
Effect of melatonin on glutamate-induced mitophagy in HT22 cells. **(A)** Fluorescence intensity of mitochondrial Mtphagy Dye and Lyso Dye in cells; **(B)** Quantitative analyses of average fluorescence intensity of mitochondrial Mtphagy Dye in cells: *F* = 26.214, *P* < 0.001; control group vs. glutamate injury group: *P* < 0.001, glutamate injury group vs. melatonin pretreatment group: *P* < 0.001; **P < 0.01. ns, not significant (*n* = 6/group).

The effect of glutamate on intracellular mitophagy was investigated under confocal microscopy. The intracellular fluorescence intensity of Mtphagy Dye in the glutamate group was higher than the control group, and melatonin pretreatment slowed the glutamate-induced increase in the intracellular fluorescence intensity of Mtphagy Dye. These results indicate that melatonin inhibited the glutamate-induced increase of mitophagy in HT22 cells (Figures 7A,B).

### Effects of Melatonin on Glutamate-Induced Changes in Mitochondrial Autophagy Protein Expression

**Figure 8:**
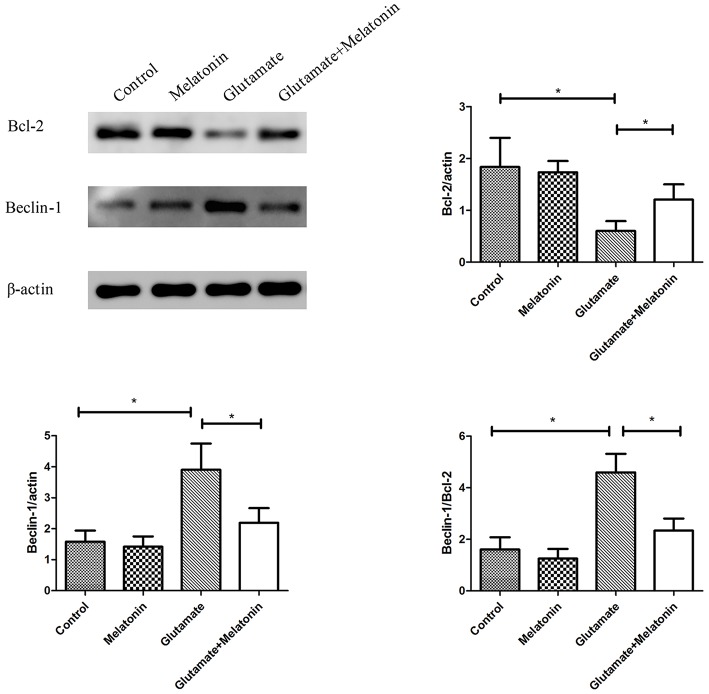
Effect of melatonin on the glutamate-induced expression of mitochondrial autophagy. Bcl-2/actin: *F* = 5.888, *P* = 0.005; control group vs. glutamate injury group: *P* = 0.010, glutamate injury group vs. melatonin pretreatment group: *P* = 0.013; Beclin-1/actin: *F* = 6.509, *P* = 0.003; control group vs. glutamate injury group: *P* = 0.011, glutamate injury group vs. melatonin pretreatment group: *P* = 0.044; Beclin-1/Bcl-2: *F* = 6.625, *P* = 0.003; control group vs. glutamate injury group: *P* = 0.010, glutamate injury group vs. melatonin pretreatment group: *P* = 0.029; *P < 0.05 (*n* = 6/group).

The effect of melatonin on the expression of mitophagy-related proteins was determined using Western blot. The results showed a decrease in the expression of Bcl-2 in the glutamate injury group and a large increase in the expression of Beclin-1 and the ratio of Beclin-1/Bcl-2 in the glutamate injury group compared to the control group. However, melatonin intervention reversed these effects, which indicates that melatonin inhibited glutamate-induced mitophagy (Figure 8, Supplementary Figures 1–3).

### The Effect of Cyclosporine A (CsA) on Glutamate-Induced Mitochondrial Autophagy

**Figure 9:**
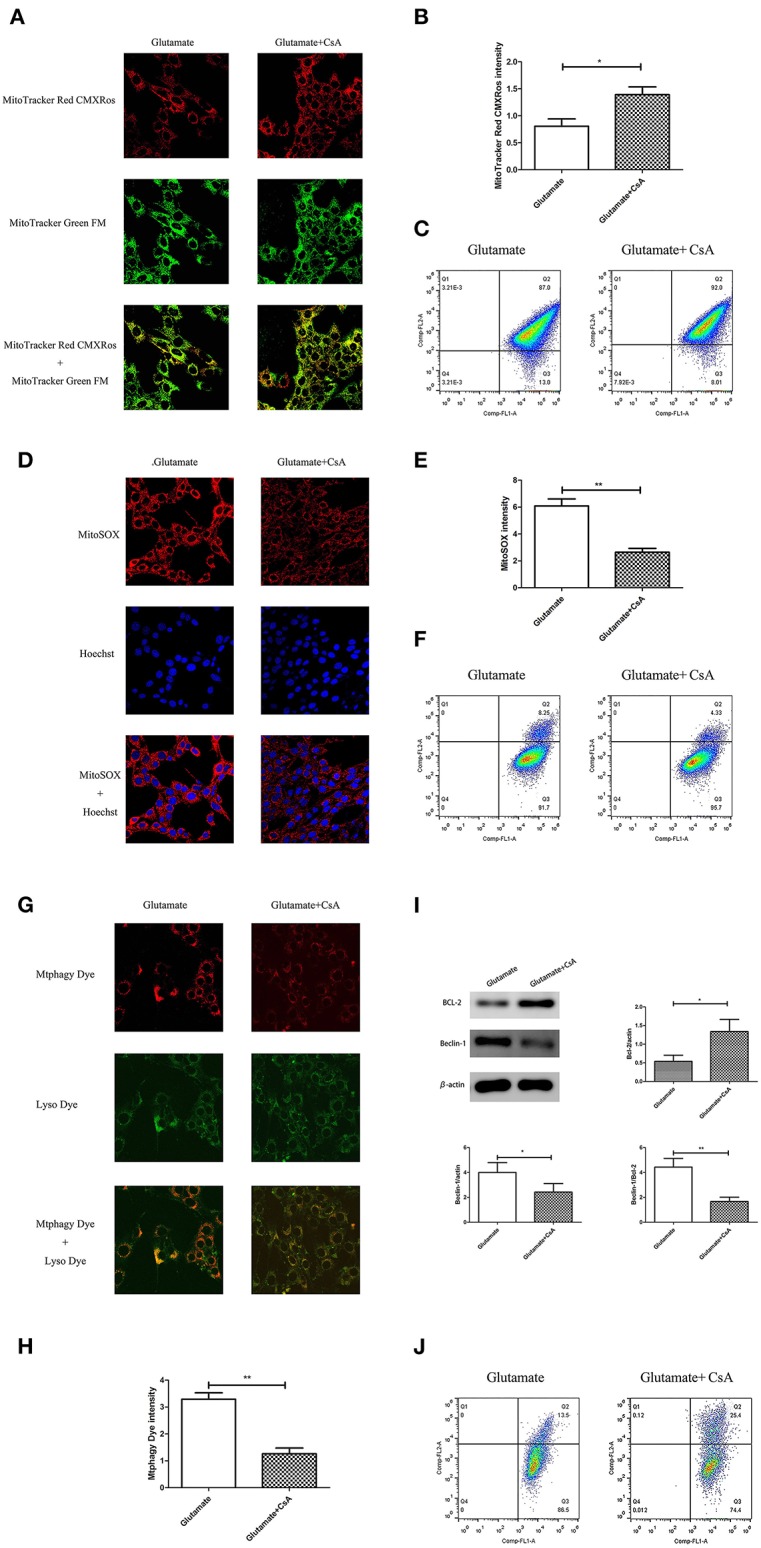
Effect of cyclosporine A (CsA) on glutamate-induced mitochondrial autophagy. **(A)** Fluorescence intensity of Mito-Tracker Red CMXROS in cells; **(B)** Quantitative analyses of mean fluorescence intensity of Mito-Tracker Red CMXROS in cells: *t* = 3.074, *P* = 0.012; **(C)** Mitochondrial membrane potential was detected using flow cytometry and Mito-Tracker; **(D)** Fluorescence intensity of mitochondrial ROS; **(E)** Quantitative analysis of mean fluorescence intensity of mitochondrial ROS: *t* = 4.511, *P* = 0.001; **(F)** Detection of ROS content in cell mitochondria using flow cytometry; **(G)** Fluorescence intensity of mitochondrial Mtphagy Dye and Lyso Dye in cells; **(H)** Quantitative analyses of mean fluorescence intensity of mitochondrial Mtphagy Dye in cells: *t* = 5.687, *P* < 0.001; **(I)** Expression of mitochondrial autophagy-related proteins in cells: Bcl-2/actin: *t* = 2.799, *P* = 0.019; Beclin-1/actin: *t* = 2.856, *P* = 0.017; Beclin-1/Bcl-2: *t* = 4.218, *P* = 0.002; **(J)** Observation of mitochondrial membrane potential changes using JC-1. *P < 0.05, **P < 0.01 (*n* = 6/group).

Cyclosporine A (CsA) is a mitophagy inhibitor. The effect of mitochondrial autophagy on glutamate-induced apoptosis was determined using Cyclosporine A (CsA). Cells were divided into a glutamate group and cyclosporine intervention group. The results indicated that CsA intervention improved the glutamate-induced red fluorescence intensity of Mito-Tracker Red CMXROS (Figures 9A,B), reversed the glutamate-induced decline of mitochondrial membrane potential (Figures 9C,J), inhibited the glutamate-induced increase of mitochondrial ROS content in cells (Figures 9D–F). slowed the increase in Mtphagy Dye fluorescence intensity in mitochondria (Figures 9G,H) and decreased the glutamate-induced expression of mitophagy-related proteins (Figure 9I). These findings indicate that melatonin inhibited glutamate-induced apoptosis via inhibition of mitophagy, which protected the cells.

## Discussion

The current study demonstrated the following principal findings: (1) glutamate resulted in mitochondrial dysfunction induced by oxidative stress in cells, which manifested in the decline of superoxide dismutase, glutathione (GSH) and mitochondrial membrane potential and a rise in GSSG (oxidized glutathione) and mitochondrial reactive oxygen species; (2) glutamate resulted in mitophagy, which was reflected by the fluorescence intensity of Mitophagy-Tracker Red CMXROS and the protein levels of Beclin-1/Bcl-2; (3) treatment with melatonin significantly restored these glutamate-induced abnormal changes; and (4) the mitophagy inhibitor CsA significantly corrected the glutamate-induced alterations, as measured by the fluorescence intensity of Mitophagy-Tracker Red CMXROS, mitochondrial ROS, and mitochondrial membrane potential changes. Our findings support the hypothesis that melatonin exerts neuroprotective effects against glutamate-induced excitotoxicity via a reduction in mitophagy-related oxidative stress and maintenance of mitochondrial function, involving the Beclin-1/Bcl-2 signaling pathway.

In this study, we used a very high glutamate concentration (5 mM). It should be pointed out that a 24 h 5 mM glutamate exposure is never going to happen *in vivo*. However, the present experiment was conducted *in vitro* model. There are many literatures that use 5 mM glutamate to treat HT22 cells to induce oxidative stress *in vitro* (42–45). In addition, our preliminary experiments treated cells with 1.25, 2.5, 5, and 10 mmol/L glutamate concentration for 24 h. The results confirmed that after 24 h of treatment with 5 mM glutamate, the cell viability was reduced to 50% or lower (data not shown). Therefore, this experiment continued to use an *in vitro* model of hippocampal neuronal damage using 5 mM glutamate used by the predecessors.

This experiment used HT22 cells, an immortalized neuronal cell line derived from the mouse hippocampus, which lacks a functional ionotropic glutamate receptor and is therefore commonly used to study non-receptor-mediated oxidation. HT22 cells have been used widely as an *in vitro* model to study the neurotoxic effects of glutamate (46, 47). It has been shown that glutamate induces HT22 mouse hippocampal cell death exclusively through the oxytotic pathway as these cells do not express functional ionotropic receptors (48). Especially, Pereira et al. (49) demonstrated that the neurotoxicity of glutamate in HT22 cells is due to the inhibition of cystine, which causes GSH consumption and oxidative stress. Especially, by using HT22 *in vitro* cell model, Herrera et al. (19) have showed that melatonin prevents cell death through a direct antioxidant effect specifically targeted at mitochondria. They found that none of the described transducers of melatonin signaling seems to be implicated in the neuroprotection provided by the indole. In addition, melatonin membrane receptors are not involved as well-known antagonists of these receptors were unable to prevent the neuroprotective effect. They found that none of the described transducers of melatonin signaling seems to be implicated in the neuroprotection provided by the indole. in addition, melatonin membrane receptors are not involved as well-known antagonists of these receptors were unable to prevent the neuroprotective effect.

In fact, melatonin has been shown to protect against a wide variety of neuronal insults and to have effects at the mitochondrial level (50), but little is known of the mechanisms implied in this effect. We recently demonstrated that exposure of HT22 cells to high doses of glutamate reduced cell survival, which was mediated by increased free radicals. We found that this mechanism involved the glutamate-induced inhibition of superoxide dismutase (SOD), glutathione (GSH) and mitochondrial membrane potential, and an increase in oxidized glutathione (GSSG) and mitochondrial ROS content (51). Here, we used MitoSOX Red Reagent and a Gallios flow cytometer to determinate mitochondrial reactive oxygen species (ROS) and the mitochondrial fluorescent probe Mito-Tracker to detect mitochondrial membrane potential. We provide additional evidence to support the findings that oxidative stress-related ROS accumulation and mitochondrial dysfunction are involved in glutamate toxicity in HT22 cells. Our present results and Takagi's findings that the decrease in mitochondrial membrane potential observed after ischemia/reperfusion injury triggered mitophagy (52) demonstrated, for the first time, that melatonin therapy reduced mitophagy-related oxidative stress and preserved mitochondria function following glutamate-induced excitotoxicity.

Autophagy is a process by which cells degrade proteins and organelles via a lysosome-dependent pathway. The pathway that degrades mitochondria via autophagy is called mitochondrial autophagy (mitophagy), which is the specific process of autophagic elimination of its own mitochondria and an important regulatory mechanism for cells to maintain homeostasis (53). Mitophagy exerts a dual function; it promotes cell survival or death depending on the cellular context and microenvironment (54). The degradation of mitochondria via the mitophagy pathway is critical for cell survival under physiological conditions (55). However, hyperactivated mitophagy impairs mitochondrial function. For example, Cesarini et al. (55) showed that melatonin supplementation reduced cell death and restored mitochondrial function via autophagy regulation in hippocampal HT22 cells from the effects of serum deprivation. Feng et al. (56) found that the myocardium was protected from ischemia/reperfusion injury via a reduction in mitochondrial dysfunction and mitophagy. The Chinese herbal extract Xiaoxuming Decoction reduced mitochondrial activation and maintained mitochondrial function via reducing the expression of LC3, Beclin-1, and Lamp1 (57). Notably, Nopparat et al. (22) demonstrated that melatonin protected the SK-N-SH neuronal dopaminergic cell line against methamphetamine- induced autophagy via inhibition of the dissociation of the Bcl-2/Beclin-1 complex. Bcl-2 plays a role in the negative regulation of autophagy by blocking Beclin-1. Here, we further validated this view by using the mitophagy inhibitor cyclosporin A (CsA). Mitochondria is considered to be the main source of ROS, and mitochondrial dysfunction can lead to impaired intracellular calcium homeostasis, induce accumulation of reactive oxygen species, and cause oxidative stress. Cyclosporin A is a specific inhibitor of mitochondrial permeability transition, which inhibits mitochondrial depolarization and mitophagy by interfering with the interaction of cyclophilin D and mitochondrial permeability transition pores. The current results showed for the first time that after the use of mitophagy inhibitor CsA, the expression of Bcl-2 was increased, the expression of Beclin-1 was decreased, the mitochondrial damage caused by glutamate was alleviated, as well as the reduced amount of released ROS, which was in accordance with the results obtained after using melatonin. We thus speculate that Bcl-2/Beclin-1 mediated mitophagy may underlying melatonin' anti-oxidative stress and neuroprotective effects induced by glutamate in an *in vitro* HT22 hippocampal cell model. The following is a graphical summary of our findings:

**Figure:**
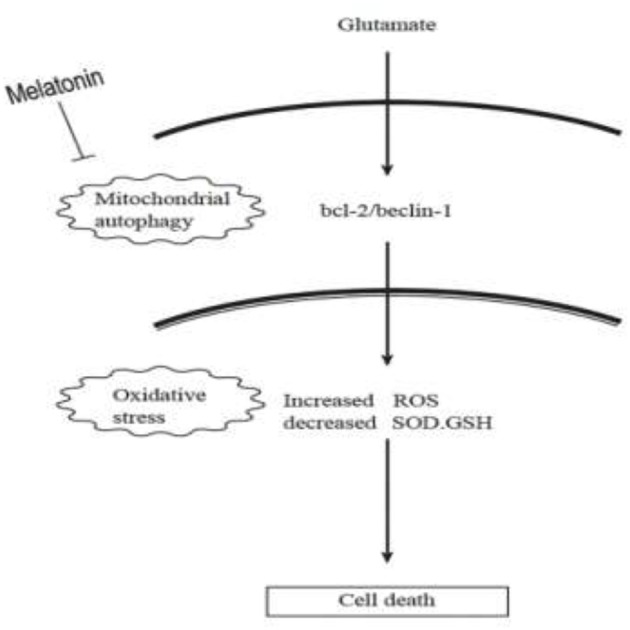


In conclusion, our findings provide new insight into the complexity of the neuroprotective role of melatonin, particularly on neuronal mitochondrial function via the mitophagy pathway after glutamate-induced neuronal excitotoxicity in HT22 cells. These findings have potential translational medical value and provide new experimental evidence for the use of melatonin as an adjunct in the treatment of epilepsy.

## Data Availability

The raw data supporting the conclusions of this manuscript will be made available by the authors, without undue reservation, to any qualified researcher.

## Author Contributions

HN was the designer and dissertation writer of this study. DW, MJ, and DZ were the operators of this experiment and were responsible for the statistical analysis of the data.

### Conflict of Interest Statement

The authors declare that the research was conducted in the absence of any commercial or financial relationships that could be construed as a potential conflict of interest.

## Supplementary Material

Supplementary Figure 1 The full-length Western blot image of β-actin protein expression in each group.

Supplementary Figure 2 The full-length Western blot image of BCL-2 protein expression in each group.

Supplementary Figure 3 The full-length Western blot image of Beclin-1 protein expression in each group.
