# Supplementary figures and images for: Reduction of Mitophagy-Related Oxidative Stress and Preservation of Mitochondria Function Using Melatonin Therapy in an HT22 Hippocampal Neuronal Cell Model of Glutamate-Induced Excitotoxicity
Source: Front Endocrinol (Lausanne). 2019 Aug 8;10:550. doi: 10.3389/fendo.2019.00550 (PMC6694460; doi:10.3389/fendo.2019.00550)

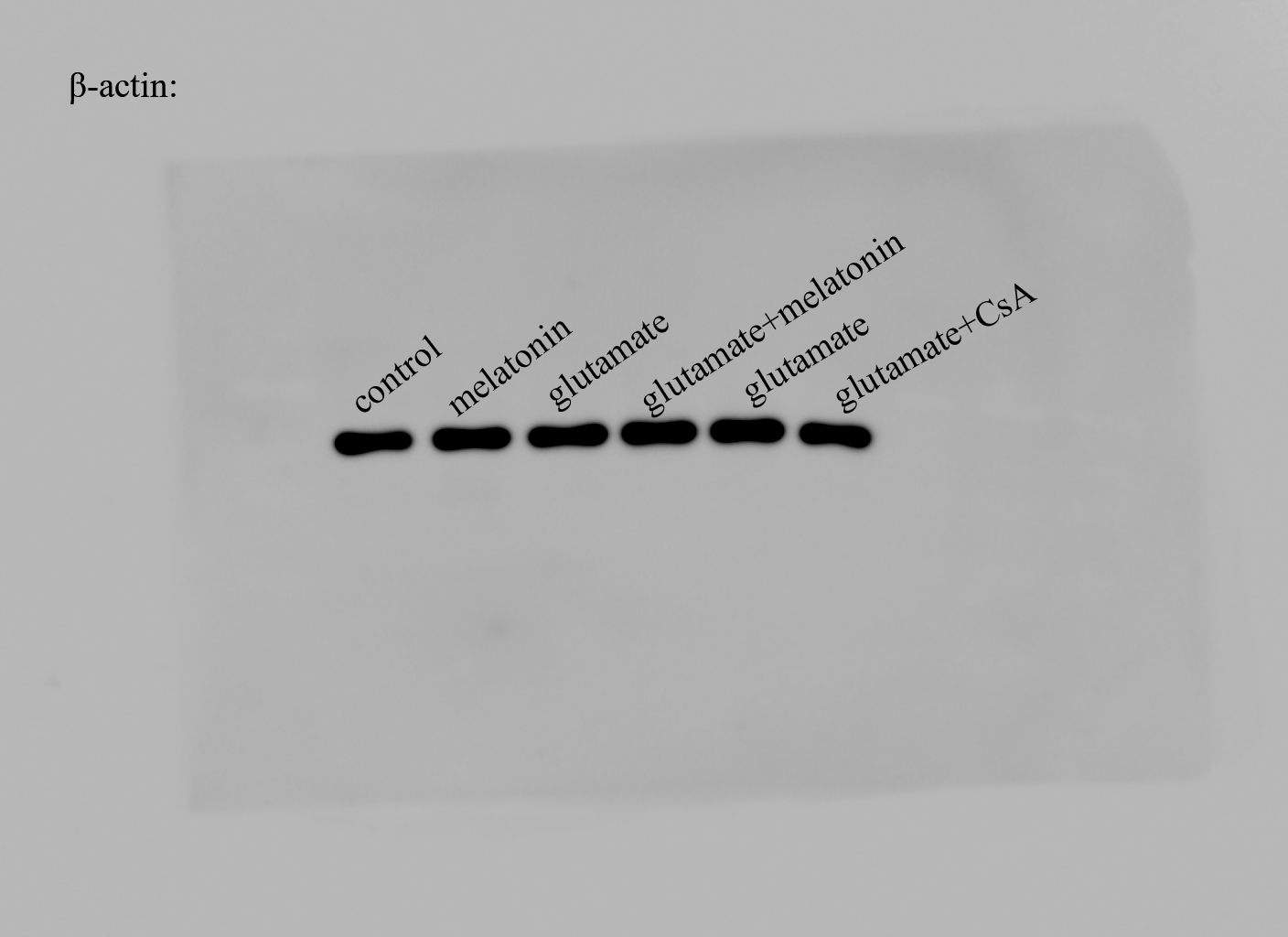

Supplement: Supplementary Figure 1 — The full-length Western blot image of β-actin protein expression in each group. [file Image_1.TIF]

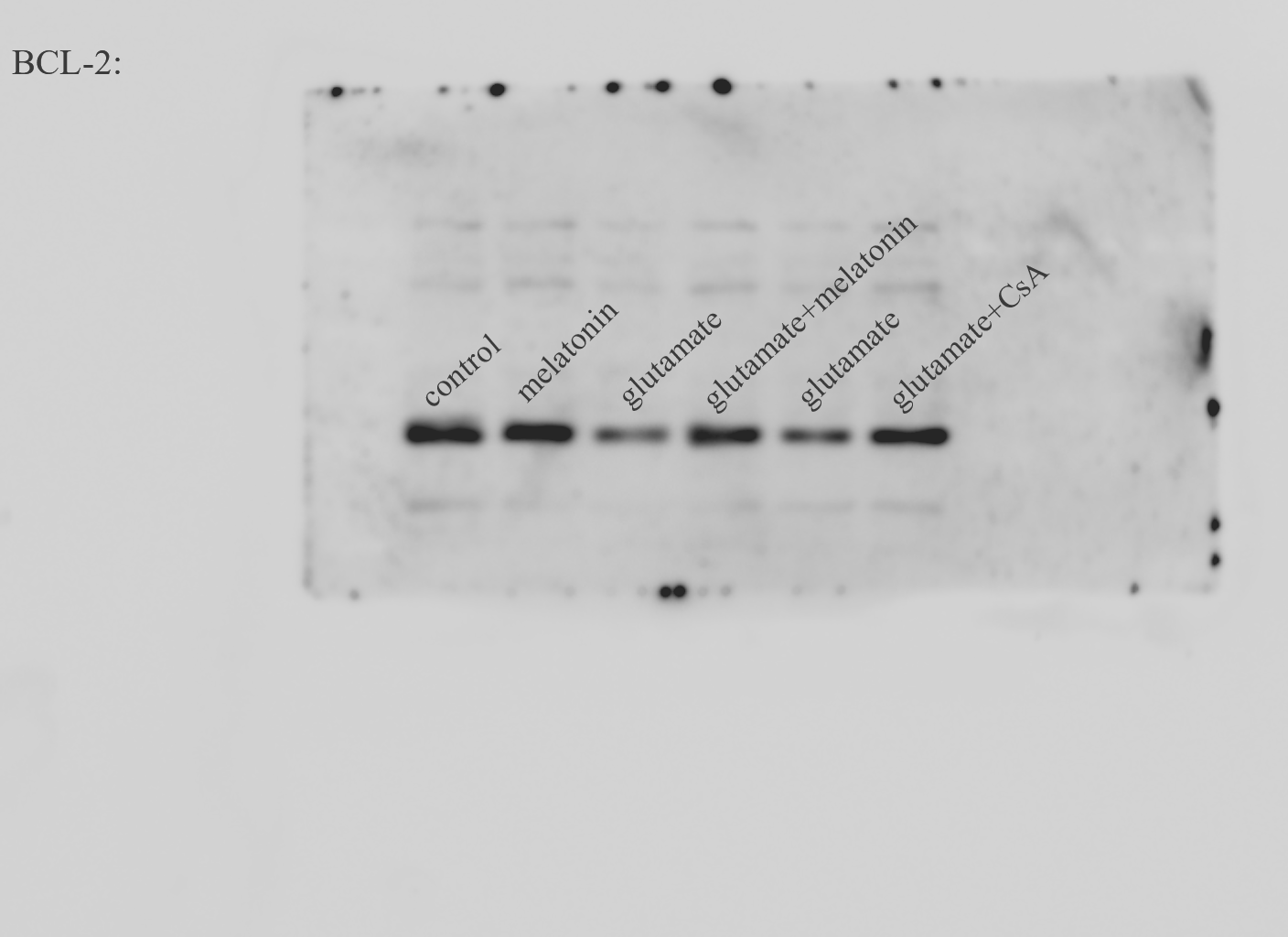

Supplement: Supplementary Figure 2 — The full-length Western blot image of BCL-2 protein expression in each group. [file Image_2.TIF]

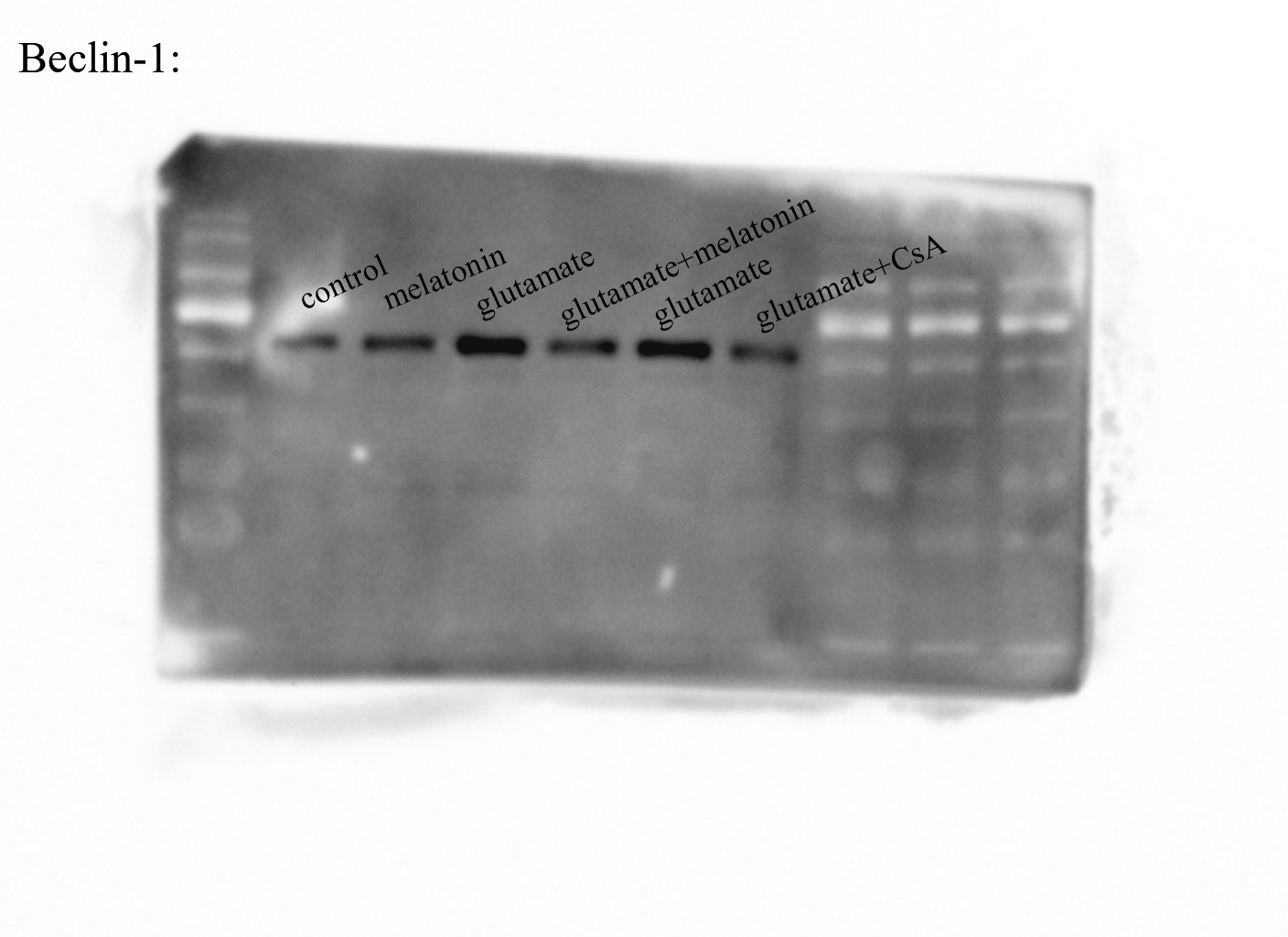

Supplement: Supplementary Figure 3 — The full-length Western blot image of Beclin-1 protein expression in each group. [file Image_3.TIF]
